# Supplementary material for: Spatial distribution and determinants of measles vaccination dropout among under-five children in Ethiopia: A spatial and multilevel analysis of 2019 Ethiopian demographic and health survey
Source: PLoS One. 2024 Jul 8;19(7):e0305393. doi: 10.1371/journal.pone.0305393 (PMC11230542; doi:10.1371/journal.pone.0305393)
Supplement: Attachment — Submitted filename: Resonse to Reviewers.docx [file pone.0305393.s005.docx]

**Response to reviewers and editor comments**

PONE-D-24-01758

**Title**: Spatial distribution and determinants of measles vaccination dropout among under-five children in Ethiopia: A spatial and multilevel analysis of 2019 Ethiopian Demographic and Health Survey

**Journal**: PLOS ONE

**Authors:** *Alebachew Ferede Zegeye, Enyew Getaneh Mekonen, Berhan Tekeba, Tewodros Getaneh Alemu, Mohammed Seid Ali, Almaz Tefera Gonete, Alemneh Tadesse Kassie, Belayneh Shetie Workneh, Tadesse Tarik Tamir, Mulugeta Wassie*

**Subject:** Submission of revised manuscript

Yours sincerely,

**Alebachew Ferede Zegeye (on behalf of all authors)**

University of Gondar, Gondar, Ethiopia

**Response to reviewers and editor comments**

We are very welcome to accept the revisions requested by the reviewers and editors of this manuscript. Please see below our detailed modifications for the manuscript (PONE-D-24-01758). Also, kindly find the attached a clean revised manuscript and a revised manuscript with tracked changes.

**Reviewer 1 Comments:**

1. The result section of the abstract should be reconsidered. For example, you say that marital status had higher odds of a measles vaccination dropout rate. Is it the single or married group that has higher odds of a drop rate? This comment also includes the factors.

- **Response**: Dear reviewer, First and foremost, thank you for your in-depth review of our manuscript and for your insightful comments. We understand the importance of clearly presenting our findings and agree that the section on marital status and its impact on measles vaccination dropout rates needs clarification. In response to your question, it was the single group that exhibited higher odds of a dropout rate in our study. We have revised the abstract accordingly to reflect this finding more explicitly. We have also reviewed the factors associated with vaccination dropout rates and ensured that each is clearly defined and its relationship to the dropout rates is unambiguously presented. Kindly see the corrections in the result section of the abstract in the revised manuscript.

2. Background section: Paragraph 2, second and third sentences convey similar idea. You should use either of one.

- **Response**: Dear Reviewer, Thank you for your observation regarding the similarity between the second and third sentences in the second paragraph of the background section. Upon re-evaluation, we agree that these sentences convey overlapping ideas and that the section’s clarity can be improved by streamlining this content. We have revised the paragraph to eliminate redundancy and enhance readability. The revised paragraph now succinctly presents the idea without repetition. We appreciate your guidance in refining our manuscript and believe that this change has improved the flow and coherence of the background section. Kindly see the amendments on page 3, line numbers 62–64.

3. Paragraph 4, first sentence is repeated a second time (1-2 million....).

- **Response**: Dear Reviewer, Thank you for bringing to our attention the inadvertent repetition of the first sentence in paragraph four. We have reviewed the section and removed the duplicate sentence to maintain the conciseness and integrity of our manuscript. Please see the amendments on page 3, line 76.

4. Methods: Study setting- latitude should be rewritten.

- **Response**: Dear reviewer, Thank you for your comment regarding the latitude details in our manuscript. We have re-evaluated the coordinates provided and acknowledge that they were incorrectly stated. We have now corrected the latitude range to accurately reflect Ethiopia’s geographical position. Kindly see the corrections in the revised manuscript on page 5, lines 120-122.

5. Study design and period: Your study is a secondary data analysis. However, you dictated as you conducted the primary data collection. I suggest this section to be rewritten.

- **Response**: Dear Reviewer, Thank you for your thoughtful feedback. We appreciate your attention to detail. Allow us to clarify the nature of our study and address your comment. Our study is indeed a secondary data analysis based on existing data from the Demographic and Health Surveys (DHS). While we did not directly collect primary data, we utilized the DHS dataset, which is a valuable resource for community-based cross-sectional studies. The DHS surveys are conducted at the community level, providing comprehensive health and demographic information across various populations.

6. Data source and sampling procedure: Binary logistic regression model and multi-level analysis model are used by the authors. How these two models applied simultaneously as the assumptions of the two models are different?

- **Response**: Dear Reviewer, Thank you for your question regarding the use of both binary logistic regression and multi-level analysis models in our study.

We understand the concern, as these models are based on different assumptions.

In our analysis, we initially applied a binary logistic regression model to explore the relationships between individual-level predictors and the outcome variable. This model allowed us to estimate the odds ratios for the likelihood of the outcome occurring given the presence of certain predictors. Subsequently, we employed a multi-level analysis model to account for the hierarchical structure of our data, where individuals are nested within larger units (e.g., communities or regions). This model enabled us to consider the variance at both the individual and group levels, which is not possible with standard logistic regression.

We believe that the sequential application of these models provides a comprehensive understanding of the factors influencing the outcome at different levels. The binary logistic regression model addresses individual-level effects, while the multi-level analysis captures group-level variations.

We hope this addresses your query and demonstrates the robustness of our analytical approach. Thank you.

7. Dependent variables: In determining the dropout rate, the descriptive sentences and the formula are not similar- should be rewritten in a way that is clear for the readers.

- **Response**: Dear reviewer, Thank you for your careful review and for pointing out the inconsistency between the descriptive text and the formula for the dropout rate in our manuscript. We appreciate the opportunity to clarify this. We have reviewed the section and understand that the discrepancy may cause confusion for readers. Although the formula provided is correct, we inadvertently described an alternative formula in the text. To resolve this, we have revised the manuscript to ensure that the descriptive sentences accurately reflect the correct formula. Kindly see the amendments on page 9, lines 236-237 of the revised manuscript.

**Reviewer 2 Comments:**

1. Immunization is the process of administering a vaccination to develop immunity against an infectious agent, with the primary goal being to prevent the disease that the infection causes (3, 4).

Comment: Change “a vaccination” to “a vaccine”. Change “with the primary goal being to prevent the disease that the infection causes” to “with the primary goal of preventing the disease caused by the infection”

- **Response**: Dear reviewer, First and foremost, we would like to express our sincere gratitude for your insightful comments and suggestions, which have significantly contributed to the improvement of this manuscript.

Regarding your comment to change “a vaccination” to “a vaccine,” we agree with this suggestion. The term “vaccine” is more precise, and we have updated the manuscript accordingly. Additionally, we have revised the phrase “with the primary goal being to prevent the disease that the infection causes” to “with the primary goal of preventing the disease caused by the infection,” as you recommended. Kindly see the changes on page 3, lines 56–57 of the revised manuscript.

2. Childhood immunization, which prevents 1-2 million childhood deaths, worldwide
   Comment: annually? specify period

- **Response**: Dear Reviewer, Thank you for your attention to detail. To clarify, the statement should indeed specify the time frame to avoid ambiguity. The revised sentence is as follows: “Childhood immunization, which prevents 1-2 million childhood deaths annually worldwide, is one of the most successful health interventions available to reduce the mortality and morbidity of infectious illnesses in children”. This change has been made on page 3, line 76 of the revised manuscript.

3. Immunization saves millions of lives annually, making it a success story for global health and development.

Comment: Given the previous sentence, this sentence is unnecessary. I suggest deleting it.

- **Response**: Dear Reviewer, Thank you for your suggestions. Upon reflection, we agree that the sentence in question may seem redundant in light of the previous statement. Therefore, we have decided to remove it to improve the conciseness and flow of the manuscript. The deletion has been made on page 3 of the revised manuscript.

4. The measles vaccine alone prevented 23 million deaths between 2010 and 2018.

Comment: Based on the following reference:

<https://www.cdc.gov/mmwr/volumes/68/wr/mm6848a1.htm> the 23 million deaths prevented refer to the 2000-2018 period

- **Response**: Dear Reviewer, Thank you for bringing this to our attention. Upon reviewing the reference you provided and cross-checking our manuscript, we realize that an editing error led to the incorrect reporting of the time frame for the measles vaccine’s impact. This correction has been made on page 3, line 79 of the revised manuscript. We are grateful for your diligence and apologize for any confusion caused by this oversight.

5. More than 116 million infants, or 86% of all babies born, receive vaccinations each year, which is an all-time high (10, 11). In sub-Saharan Africa, 4.4 million children lose their lives due to communicable diseases each year as a result of inadequate vaccination and setup, incomplete vaccination, and impediments to delivering vaccinations, although these deaths may have been avoided by vaccination (12, 13).

Comment: Given the previous sentence, the focus is already on the measles vaccine. Why go back on all vaccines in general? Isn't that complementary to what was written above (“Each year, vaccine-preventable diseases such as … incomplete vaccination coverage (6-8).”)?

- **Response**: Dear Reviewer, We thank you for your comment and for prompting us to clarify the scope of our discussion on vaccines. The sentence in question aims to provide a broader context for the success of vaccination programs globally, highlighting the high coverage of infant immunization. We believe this sets the stage for the subsequent discussion on the specific challenges faced in sub-Saharan Africa, which contrasts the global success with regional struggles.

However, we understand your concern that this may seem redundant given the previous focus on the measles vaccine. To maintain the manuscript’s coherence and focus, we have revised the text to better integrate the global perspective with the specific issues related to measles vaccination in sub-Saharan Africa. The amendment is found on page 3, lines 78-84 of the revised manuscript. We appreciate your guidance in ensuring that our manuscript presents a clear and focused narrative.

6. Although all children are expected to receive the measles vaccination at nine months, in Ethiopia, as part of the government's subsidized programme, 3.4% of children did not receive complete measles virus immunization through routine immunization services in 2015 (14).

Comment: Please provide more details on measles incidence, case and mortality estimates, and vaccine coverage in the African region, if possible, and in Ethiopia in particular. Examples of how other authors have done this may be found here:

<https://www.cdc.gov/mmwr/volumes/72/wr/mm7236a3.htm?s_cid=mm7236a3_w>.

- **Response**: Dear Reviewer, Thank you for your request for additional details regarding measles incidence, case and mortality estimates, and vaccine coverage. We have updated our manuscript to include the most recent and relevant data available. These updates have been incorporated into the revised manuscript on page 3, lines 63-67 and lines 69-75, and we believe they provide a comprehensive overview of the current measles situation in the African region and Ethiopia specifically.

7. A measles vaccination dropout occurs when a child has received the first recommended dose of the vaccine but has missed the second recommended measles vaccination dose.

Comment: It would be much more relevant to show the gap between MCV1 and MCV2 before introducing the concept of vaccine dropout.

- **Response**: Dear reviewer, Thank you for your insightful comments and suggestions regarding our manuscript. We agree that understanding the interval between the first and second doses of the measles vaccine is crucial for comprehensively addressing the issue of vaccine dropout. To this end, we have revised our manuscript to include a detailed analysis of the coverage gap between the administration of the first dose (MCV1) and the second dose (MCV2) of the measles vaccine. Kindly see the amendments on page 4, lines 91-96 of the revised manuscript.

8. Vaccination dropout is used as an indicator of immunization program performance, and low dropout rates indicate good access and utilization of immunization services

Comment: Add “to” after good access

- **Response**: Dear reviewer, Thank you for your attention to detail in reviewing our manuscript. We have taken your suggestion into account and have made the correction. Kindly see the amendments on page 4, line 100 of the revised manuscript.

9. If they are more than 10%, there is typically a major quality issue that has to be addressed (15, 18).

Comment: For consistency, this sentence needs to follow: “Vaccination dropout is used as an indicator of immunization program performance, and low dropout rates indicate good access to and utilization of immunization services (17)”.

- **Response**: Dear reviewer, Thank you for your comment emphasizing the importance of consistency in our manuscript. Please see the amendment on page 4, lines 98-100 of the revised manuscript.

10. Despite achievements observed in the reduction of under-five mortality rates, about 1-2 million children are still dying each year due to vaccine-preventable diseases, and sub Saharan African countries share the huge burden of global under-five mortality.
    Comment: This point has already been discussed above. What is the point of mentioning it again?

- **Response**: Dear reviewer, Thank you for your observation. In the revised manuscript, we have removed the previous mention of the under-five mortality rates to avoid repetition. Kindly see the amendment on page 4, line 107 of the revised manuscript.

11. As far as our search of the literature and knowledge goes, the risk (hotspot) areas for measles vaccination dropout among under-five children have not been identified in Ethiopia.

Comment: What about: https://www.mdpi.com/2076-393X/12/3/328? How is this study different from yours?

- **Response**: Dear reviewer. Thank you for directing us to the study published in MDPI. The referenced study conducted a cross-sectional survey using primary data from mothers/caregivers in Ethiopia’s underprivileged areas, focusing on spatial analysis of high measles vaccination dropout. Our study, however, utilizes secondary data from the Demographic and Health Surveys (DHS), which allows for a broader analysis across various regions and time periods and allows us to consider individual and community-level factors affecting measles vaccination dropout across the entire country. While the above study provides valuable insights into specific high-risk areas, our research offers a comprehensive overview of national trends and factors associated with measles vaccination dropout. The use of DHS data enables us to identify systemic issues and potential interventions that can be applied on a national scale, complementing the findings from more localized studies like the one you mentioned.

Methods:

12. Data source and sampling procedure: The Ethiopian Demographic and Health Survey (EDHS 2019) data was used to investigate the spatial distribution of measles vaccination dropouts and determinates among under-five children

Comment: Do you mean determinants among under-five children?

- **Response**: Dear reviewer, Thank you for your careful reading and for pointing out this typographical error. You are correct; the intended word is ‘determinants.’ We have revised the manuscript to reflect this correction. We appreciate your attention to detail, which has helped improve the clarity and accuracy of our study. Kindly see the correction on page 6, line 145.

13. A binary logistic regression model was used to determine the factors associated with measles vaccination dropout rates. Determinants of the measles vaccination dropout were reported in terms of an adjusted odds ratio (AOR) with a significance level of 95%. In the univariable analysis, at 95% confidence intervals, a p-value of <0.25 was considered a candidate for the multivariable analysis of the data. All variables with p-values <0.05 were considered statistically significant in multivariable logistic regression.

Comment: This part should not be in the data source and sampling procedure section

- **Response**: Dear reviewer, Thank you for your comment. We agree that the details of the binary logistic regression model are more suited to the multilevel statistical analysis section of our manuscript. We have made the necessary changes, and this information can now be found under the heading ‘Multilevel Analysis.’ We appreciate your guidance in improving the organization and clarity of our study. Kindly see the correction on page 7, lines 165-170.

14. Spatial analysis: Using Getis-OrdGi* statistics, significant “hot-spot” and “colds-pot” areas for measles vaccination dropout have been identified.

Comment: Please provide arguments justifying the use of Getis-OrdGi* statistics instead of local Moran’s I (LISA) statistic measures in your case

- **Response**: Dear reviewer, Thank you for your question regarding our choice of statistical measures. We chose to use Getis-Ord Gi* statistics for several reasons that align with the objectives of our spatial analysis. Firstly, Getis-Ord Gi* is particularly effective for identifying hot-spot and cold-spot areas with high precision, which is essential for our study’s focus on measles vaccination dropout. This method allows us to pinpoint specific locations where intervention is most needed. Secondly, Getis-Ord Gi* provides a clear distinction between hot-spots and cold-spots, which is crucial for our analysis as it directly informs targeted public health strategies. Lastly, while local Moran’s I (LISA) is also a robust measure for spatial autocorrelation; it tends to be more generalized and less sensitive to subtle variations in small-scale spatial patterns, which are significant in our study. Therefore, Getis-Ord Gi* was deemed more suitable for our specific research goals and the nature of our data. Please see the amendments on page 8, lines 205-213.

15. Study variables: The measles vaccination dropout rate was calculated by dividing the number of children aged 0-59 months who received measles 1 by the number of children aged 0-59 months who received measles 2 divided by the number of children 0-59 months of age who received measles 1 multiplied by 100%;

Comment: The formula does not match the given operational definition of measles vaccination dropout.

- **Response**: Dear reviewer, Thank you for your attention to the accuracy of our study variables. Even though the previous formula was possible to calculate measles vaccination dropout rate we have revised the formula to avoid confusion for calculating the measles vaccination dropout rate to ensure it aligns with the operational definition. Kindly see corrected formula on page 9, line 236-237.

16. Birth interval (months) (<24, 24-28, >48)

Comment: Do you mean <24, 24-48, >48?

- **Response**: Dear Reviewer, Thank you for bringing this to our attention. You are correct; there was a typographical error in the categorization of the birth intervals. The intended categories should be <24 months, 24-48 months, and >48 months. We apologize for any confusion this may have caused and appreciate your diligence in reviewing our work. Please see the corrections on page 9, line 241.

17. Number of under-5 children (≤1, 2, ≥3)

Comment: ≤1 means 0 to 1. Can you explain how it is possible to drop out of measles vaccination if there are 0 children under 5 years old?

- **Response**: Dear Reviewer, Thank you for your insightful comment. The category “≤1” was intended to include households with one child under the age of 5, as well as those with no children in this age group. We realize this may have caused some confusion. To clarify, households with 0 children under 5 years old would naturally not be included in the analysis of measles vaccination dropout rates. Our focus was on households with at least one child eligible for vaccination.

18. Random effects (Measures of variation): Moreover, the PCV

Comment: Change “PCV” to “proportional change in variance (PCV)”

- **Response**: Dear Reviewer, Thank you for your recommendation to spell out the abbreviation “PCV” in our manuscript. We have amended the text to read “proportional change in variance (PCV)” as suggested. This change has been made on page 11, line 285.

Results

19. Hotspot analysis of measles vaccination dropout: The local Getis-Ord Gi* statistics were employed in the present study to determine the measles vaccination dropout hot and cool spots.

Comment: Change “cool” to “cold”

- **Response**: Comment accepted, dear reviewer; we appreciate your attention to detail and your suggestion to use the term “cold spots” instead of “cool spots” for consistency and clarity in the context of our hotspot analysis. We have reviewed our manuscript and made the necessary corrections as recommended. Please see the correction on page 14, line 345.

20. The odds of measles vaccination dropout rates were 1.87 times higher among under-five children whose birth interval was less than 24 months compared to under-five children whose birth interval was greater than 48 months

Comment: Change “compared to” to “compared with”

- **Response**: Dear Reviewer, Thank you for your suggestion to improve the clarity of our comparative statement. We have updated the manuscript to reflect the change from “compared to” to “compared with” as you recommended. This correction has been applied to all relevant instances within the document. Kindly see the correction on page 17, line 404.

21. Measles vaccination dropout rates were 3.98 times higher among under-five children born from unmarried mothers as compared to under-five children delivered from mothers who had married

Comment: Change to “among children under five born to unmarried mothers than among children under five born to married mothers”

- **Response**: Dear Reviewer, We have accepted your suggestion for rephrasing our statement for better clarity. We believe this change improves the readability and precision of our findings. Kindly see the amendments on page 17, lines 405-407 of the revised manuscript. Thank you for your constructive feedback.

22. The odds of measles vaccination dropout rates were 3.86 times higher among under five children, where the number of under-five children is one and less compared to the number of under-five children of three or more

Comment: Change “compared to” to “compared with”

- **Response**: Dear Reviewer, We have taken your suggestion into consideration and have revised the manuscript accordingly. Please see the correction on page 17, line 404 of the revised manuscript. Thank you for your guidance in enhancing the precision of our language.

23. I suggest to the authors to merge table 3 and table 4. Examples of how other authors have done this may be found in the following articles:

https://bmcpublichealth.biomedcentral.com/articles/10.1186/s12889-015-2315-z

<https://bmcpublichealth.biomedcentral.com/articles/10.1186/s12889-018-5881-z>

- **Response**: Dear Reviewer, We have considered your suggestion to merge Table 3 and Table 4 and found it to be beneficial for the clarity and conciseness of our presentation. We have combined the tables as recommended, which has allowed us to present our data more effectively. Kindly see the corrections in Table 3.

Discussion

24. Despite a modest improvement in vaccination rates globally from 2021 to 2022, 33 million children were still not vaccinated against measles: around 22 million missed their first dose, and an additional 11 million, or 74% of the total, missed their second dose (30).

Comment: Please also provide details on children missing measles vaccine doses in Ethiopia.

- **Response**: Dear Reviewer, We have amended our manuscript to include the requested details on the measles vaccination coverage among children in Ethiopia. The updated section now provides comprehensive data on the number of children who have missed their first and second doses of the measles vaccine. kindly see the amendment on page 20, lines 440-444.

25. In this study, the prevalence of measles vaccination dropout among under-five children in Ethiopia was found to be 33.26% (95% CI: 31.65, 34.91).

Comment: Not necessary to add: “(95% CI: 31.65, 34.91)”

- **Response**: Dear Reviewer, Based on your recommendation, we have removed the 95% confidence interval (CI) from the reported prevalence of measles vaccination dropout among under-five children in Ethiopia. Please see the correction on page 20, line 447. Thank you for your guidance on this matter.

26. The potential explanation for these discrepancies may be due to differences in health policy, quality of health care, and socioeconomic and cultural differences across the countries.

Comment: Please provide references to support your assumptions.

- **Response**: Dear Reviewer, Thank you for your comment requesting references to support our assumptions about the differences in measles vaccination dropout rates across various countries. We have reviewed the literature and included relevant studies that discuss the factors influencing these rates. Kindly see reference numbers 43, 48, and 50 on page 20 of the revised manuscript.

27. The spatial autocorrelation statistic confirmed that the distribution of measles vaccination dropout was clustered in some geographical areas of Ethiopia (Moran’s I = 0.358552, p <0.001).

Comment: No need to add: “(Moran’s I = 0.358552, p <0.001)”.

- **Response**: Dear Reviewer, We have taken into account your suggestion to remove the specific Moran’s I value and the associated p-value from our discussion of the spatial autocorrelation statistic on page 20, line 459. Thank you for your recommendation to streamline our presentation.

28. The most plausible explanation for this geographical variance in measles vaccine dropout rates could be due to significant differences in infrastructure, such as the distribution of healthcare facilities and professionals among regions, as well as differences in roads, electricity, and water.

Comment: Please provide references to support your assumptions.

- **Response**: Dear Reviewer, Thank you for your comment. We acknowledge the importance of substantiating our assumptions with empirical evidence. To support our discussion on the geographical variance in measles vaccine dropout rates, we have included references that explore the impact of infrastructure on health outcomes. Kindly see references 52 and 53 on page 20 of the revised manuscript.

29. The possible explanation might be that short birth intervals can cause a burden on a woman's ability to care for her dependent children as well as other responsibilities in the home. Because of this, mothers might not have the time or desire to bring their children to the health institution to get essential vaccines.

Comment: Please provide references to support your assumptions.

- **Response**: Dear Reviewer, Thank you for your comment. We agree that providing empirical evidence is crucial to supporting our assumptions. To substantiate our claim regarding the impact of short birth intervals on vaccination rates, we have included references that discuss the association between birth intervals and child health outcomes, including vaccination coverage. Kindly see references 57 and 58 on page 21 of the revised manuscript.

30. The possible explanation could be that a large family size is likely to strengthen maternal capabilities to extend more care to the younger children as well as her mobility to get access to immunization services due to experience.

Comment: Please provide references to support your assumptions. How can you explain the observed discrepancies between your findings and those of the studies conducted in Ghana, Australia, India, Lebanon, and Brazil?

- **Response**: Dear Reviewer, Thank you for your insightful comments. To support our assumptions regarding the positive impact of large family size on maternal capabilities and access to immunization services, we have included references that discuss the benefits of extended family support systems and the experience gained from managing larger families. Please see reference 67 on page 22 of the revised manuscript.

Regarding the discrepancies between our findings and those from studies conducted in Ghana, Australia, India, Lebanon, and Brazil, we have conducted a thorough review of the literature to understand the context-specific factors that may contribute to these differences. Please see the amendments on page 22, lines 501-503.

31. Measles vaccination dropout was 2.43 times higher among under-five children whose place of residence was rural as compared to under-five children from urban residence. This is consistent with the findings from previous studies in Malawi (55). The possible explanation might be that the low vaccination dropout rate among urban children may be due to the following factors: highly educated mothers may have greater control and autonomy over residence resources; they may also have changed traditional perspectives about immunization. As a result, they might be more likely to seek healthcare and acquire new information about health issues earlier.

Comment: There is substantial evidence that children living in rural areas are more likely to drop out measles vaccination than children living in urban areas. Please provide more references. Provide also references to support your assumptions.

- **Response**: Dear Reviewer, Thank you for your request for further references to support our findings and assumptions. We have incorporated additional literature that substantiates the higher likelihood of measles vaccination dropout among children in rural areas compared to their urban counterparts. Kindly see the amendments on page 22, lines 511-114, and references 53 and 78.

32. The findings of this study are contradictory to those of the studies conducted in India (59) and Bangladesh (60). The possible explanation for the inconsistency might be Low vaccination dropouts in rural areas are due to the use of traditional birth attendants and primary health care workers, who both play a role in encouraging mothers to attend the maternal and child health clinics, although these roles do not formally exist in urban areas.

Comment: It would be much more relevant to mention other studies conducted in African settings. Provide also references to support your assumptions.

- **Response**: Dear Reviewer, Thank you for your valuable feedback. We agree that it is essential to consider studies conducted within similar contexts when discussing our findings. To address your comment, we have included references from research conducted in African settings that examine the role of traditional birth attendants (TBAs) and primary health care workers in influencing vaccination rates. Kindly see the references 41, 79, 80, and 81 on page 22, lines 515-516.

33. The odds of measles vaccination dropout rates were 3.20 times higher among under-five children who have low community level ANC utilization as compared to under-five children whose community level ANC utilization was high. This finding is in line with other studies in Ethiopia (63) and Gambia (33). This association might be a result of the fact that mothers who do not make full use of ANC services miss out on opportunities to get information about the importance and timing of child vaccinations. In addition, mothers who decide not to receive antenatal care or give birth in a healthcare facility might not give the same attention to childhood vaccinations as their peers from similar socioeconomic backgrounds.

Comment: Please provide references to support your assumptions.

- **Response**: Comment accepted, Dear Reviewer, Thank you for your thoughtful feedback. We appreciate your attention to detail. We have provided references to support our assumptions. Kindly see references 87–89 on page 23, line 531.

34. Under-five children whose residence is in the Benishangul Gumuz region have a 1.80 times higher measles vaccination dropout rate compared to the Amhara region. The possible explanation for the association might be that even though vaccinations are free in Ethiopia, mothers must still cover certain indirect costs (such as food and transportation expenses), which frequently restrict their use of maternity and child health services, including immunization. In this sense, mothers in the lower income category are more likely to encounter these obstacles when trying to get to healthcare services, which eventually results in immunization dropouts.

Comment: Please provide references to support your assumptions.

- **Response**: Dear Reviewer, Thank you for your insightful comments and for prompting us to provide additional references to support our assumptions. We agree that substantiating our claims strengthens the manuscript and appreciate the opportunity to do so. Kindly see references 90 and 91 on page 23, lines 538-539.

35. Strength and Limitations of the study

Comment: Strengths and limitations of the study are clearly described. However, strengths of the EDHS may be more detailed and explicit. In addition, more about limitations to generalize information may be more explicit, vast and comprehensive.

- **Response**: Thank you for your constructive feedback on our manuscript. We appreciate your recognition of the study’s strengths, particularly our use of the recent large-sample national demography and health survey and the application of mixed multilevel logistic regression analysis. We have added details on the strength and limitations, please see the details on page 23, lines 544-546, and lines 552-555

36. Conclusions and recommendations

Comment: Given the relevance of the findings, the conclusions and recommendations are rather laconic.

- **Response**: We are grateful for your feedback and the opportunity to enhance the depth of our study’s conclusions and recommendations. Recognizing the critical nature of our findings, we have expanded these sections to more accurately reflect the urgency and importance of addressing measles vaccination dropout rates in Ethiopia. Kindly see the amendments on page 24 lines 565-568
