# Supplementary material for: Spatial distribution and determinants of measles vaccination dropout among under-five children in Ethiopia: A spatial and multilevel analysis of 2019 Ethiopian demographic and health survey
Source: PLoS One. 2024 Jul 8;19(7):e0305393. doi: 10.1371/journal.pone.0305393 (PMC11230542; doi:10.1371/journal.pone.0305393)
Supplement: Attachment — Submitted filename: Resonse to Reviewers.docx [file pone.0305393.s006.docx]

**Response to reviewers and editor comments**

PONE-D-24-01758

**Title**: Spatial distribution and determinants of measles vaccination dropout among under-five children in Ethiopia: A spatial and multilevel analysis of 2019 Ethiopian Demographic and Health Survey

**Journal**: PLOS ONE

**Authors:** *Alebachew Ferede Zegeye, Enyew Getaneh Mekonen, Berhan Tekeba, Tewodros Getaneh Alemu, Mohammed Seid Ali, Almaz Tefera Gonete, Alemneh Tadesse Kassie, Belayneh Shetie Workneh, Tadesse Tarik Tamir, Mulugeta Wassie*

**Subject:** Submission of revised manuscript

Yours sincerely,

**Alebachew Ferede Zegeye (on behalf of all authors)**

University of Gondar, Gondar, Ethiopia

**Response to reviewers and editor comments**

We are very welcome to accept the revisions requested by the reviewers and editors of this manuscript. Please see below our detailed modifications for the manuscript (PONE-D-24-01758). Also, kindly find the attached a clean revised manuscript and a revised manuscript with tracked changes.

**Journal Requirements:**

1. Please review your reference list to ensure that it is complete and correct. If you have cited papers that have been retracted, please include the rationale for doing so in the manuscript text, or remove these references and replace them with relevant current references. Any changes to the reference list should be mentioned in the rebuttal letter that accompanies your revised manuscript. If you need to cite a retracted article, indicate the article’s retracted status in the References list and also include a citation and full reference for the retraction notice.

- **Response**: Dear editor, Thank you for your thorough review of our manuscript. We appreciate your attention to detail and your commitment to maintaining the integrity of scientific literature. Upon reviewing our reference list, we confirm that we have not cited any retracted papers. Our selection of references is based on current and relevant sources, ensuring the accuracy and reliability of the information presented in our manuscript. In light of your feedback, we have carefully examined each reference to ensure completeness and correctness. All necessary details, including author names, publication years, titles, journal/book names, volume, issue, page numbers, and DOIs (where applicable), have been meticulously verified.

**Reviewer comments**

1. Introduction: For greater consistency, the paragraph on “The interval between the first and … community poverty (19-21)” and the one on “A measles vaccination dropout occurs … place of residence (24, 25)” should be merged and rewritten as they are complementary.

- **Response**: Dear reviewer, Thank you for your guidance on improving the consistency of our manuscript. We have merged and rewritten the paragraphs concerning the interval between the first and second doses of the measles vaccine (MCV1 and MCV2) and the issue of measles vaccination dropout rates. We appreciate your attention to detail and are confident that these revisions have strengthened the paper. Please find the revised manuscript on page 4, lines 92-101.

2. Discussion: The potential explanation for these discrepancies may be due to differences in health policy, quality of health care, and socioeconomic and cultural differences across the countries.

Comment: References were provided to support the hypotheses. However, it does not seem sufficient to reflect the studies conducted in Nigeria and Ghana on the one hand and Gambia, South Africa and India on the other. It is necessary to be more explicit to emphasize the factual similarities and differences based on health policy, quality of health care, and socioeconomic and cultural characteristics.

- **Response**: Dear Reviewer, Thank you for your insightful feedback. We appreciate your attention to the potential discrepancies in our study. In response, we have made the necessary revisions to enhance the clarity and specificity of our discussion. We have expanded our reference list to include studies conducted in Nigeria and Ghana, as well as those from Gambia, South Africa, and India. By doing so, we aim to provide a more comprehensive foundation for our analysis. Additionally, we have explicitly emphasized the factual similarities and differences based on health policy, quality of health care, and socioeconomic and cultural characteristics across the countries under investigation. Kindly see the amendments on page 20, lines 445-448 and lines 455-462.
